# Avian Metapneumovirus: A Narrative Review of Biology, Epidemiology, Transmission Ecology, Diagnostics and Control with Special Reference to Africa and the Middle East

**DOI:** 10.3390/vetsci13070668

**Published:** 2026-07-09

**Authors:** Omar S. Saeed, Sara A. Shabana, Mahmoud Gamal, Basem M. Ahmed, Ayman H. El-Deeb, Haitham M. Amer

**Affiliations:** 1 Department of Virology, Faculty of Veterinary Medicine, Cairo University, Giza 12211, Egypt; basem-ahmed@cu.edu.eg (B.M.A.); ayman_vv@cu.edu.eg (A.H.E.-D.); hamoamer@cu.edu.eg (H.M.A.); 2 Independent Researcher, Giza 12211, Egypt; vet.sara.alaa@gmail.com; 3 Department of Biochemistry and Molecular Biology, Faculty of Veterinary Medicine, Cairo University, Giza 12211, Egypt; mahmoud.gamal@cu.edu.eg; 4 Faculty of Veterinary Medicine, King Salman International University, El-Tor 46618, Egypt; 5 Faculty of Veterinary Medicine, Egyptian Chinese University, Cairo 11437, Egypt

**Keywords:** avian metapneumovirus, aMPV, subtype B, wild birds, wildlife–poultry interface, Africa

## Abstract

Avian metapneumovirus (aMPV) is an important respiratory pathogen of poultry associated with respiratory disease, reproductive losses, secondary bacterial complications, and economic losses in commercial poultry systems worldwide. This narrative review summarizes current evidence regarding the epidemiology, molecular diversity, transmission ecology, diagnosis, vaccination, and wildlife–poultry interactions of aMPV across Africa and the Middle East. This literature was identified through structured searches of PubMed, Scopus, Web of Science, Google Scholar, and ScienceDirect using predefined keywords related to epidemiology, molecular characterization, surveillance, wild birds, diagnostics, and vaccination. Available evidence demonstrates widespread endemic circulation of aMPV, with subtype B predominating across most investigated poultry systems. However, major heterogeneity exists among studies regarding surveillance intensity, diagnostic methodologies, molecular characterization, and reporting standards. Current evidence suggests possible involvement of wild birds in viral dissemination, although definitive reservoir competence and transmission directionality remain insufficiently established. Important surveillance gaps persist across several African and Middle Eastern countries, emphasizing the need for harmonized surveillance, subtype-matched vaccination strategies, and integrated regional monitoring.

## Simple Summary

Avian metapneumovirus (aMPV) remains an important respiratory pathogen of poultry associated with substantial economic losses in commercial turkey and chicken production systems worldwide. Current evidence demonstrates widespread circulation of predominantly aMPV-subtype B strains across Africa and the Middle East. This review summarizes available evidence regarding the molecular epidemiology, transmission ecology, diagnosis, vaccination, and control of aMPV, including the possible epidemiological role of wild birds at wildlife–poultry interfaces. Although molecular surveillance and diagnostics have improved considerably, important gaps remain regarding viral ecology, interspecies transmission, and regional surveillance coverage. Continued molecular monitoring, integrated surveillance, improved biosecurity, and vaccination strategies remain essential for long-term aMPV control.

## 1. Introduction

Avian metapneumovirus (aMPV) is an important respiratory pathogen affecting commercial poultry worldwide, particularly turkeys and chickens. The virus is associated with respiratory disease, reduced productive performance, impaired reproductive efficiency, and increased susceptibility to secondary bacterial infections, resulting in substantial economic losses in intensive poultry production systems. In turkeys, aMPV is the primary etiological agent of Turkey Rhinotracheitis (TRT), whereas in chickens it is commonly associated with Swollen Head Syndrome (SHS) [1].

Since its initial emergence, aMPV has become widely distributed across major poultry-producing regions including Europe, Asia, Africa, Middle East, and Americas. The epidemiology of the virus has become increasingly complex because of the circulation of genetically diverse strains, intensification of poultry production systems, poultry trade, and variability in vaccination and biosecurity practices. Among the recognized lineages, subtype B currently predominates in many investigated poultry-producing regions worldwide [2,3].

Accordingly, this review provides a structured narrative synthesis of the current evidence regarding the epidemiology, molecular diversity, transmission ecology, diagnosis, vaccination, and wildlife–poultry interactions of aMPV across Africa and the Middle East regio.

## 2. Review Methodology

### 2.1. Search Strategy

A structured literature search was conducted using PubMed, Scopus, Web of Science, Google Scholar, and ScienceDirect databases to identify publications related to avian metapneumovirus (aMPV). The search covered studies published from 1971 and April 2026.

Search terms included combinations of: “avian metapneumovirus”, “aMPV”, “avian pneumovirus”, “subtype A”, “subtype B”, “subtype C”, “molecular epidemiology”, “wild birds”, “migratory birds”, “transmission ecology”, “respiratory disease”, “surveillance”, “vaccination”, “phylogenetic analysis”, and “wildlife–poultry interface”. Boolean operators (“AND”, “OR”) were applied to refine retrieval sensitivity and identify studies relevant to Africa, the Middle East, and other major poultry-producing regions.

Representative search combinations included:

(“avian metapneumovirus” OR “aMPV”) AND (“epidemiology” OR “surveillance”); (“avian metapneumovirus”) AND (“wild birds” OR “migratory birds”); (“aMPV”) AND (“vaccination” OR “transmission ecology” OR “phylogenetic analysis”).

### 2.2. Eligibility Criteria

Studies were included if they met the following criteria: (i) peer-reviewed publications available in English; (ii) investigations involving avian metapneumovirus (aMPV) in poultry or wild birds; (iii) epidemiological, molecular, diagnostic, pathological, ecological, surveillance, or vaccine-related studies; and (iv) studies directly relevant to the objectives and thematic scope of this review.

Relevant review articles and reports issued by international animal health organizations were additionally considered where appropriate to provide contextual interpretation and background information.

Studies were excluded if they were duplicate publications, conference abstracts lacking sufficient methodological detail, inaccessible full texts, non-English publications, studies not directly related to aMPV, publications with insufficient epidemiological or methodological information, or articles that did not contribute substantially to the predefined review objectives.

### 2.3. Study Selection, Data Organization, and Synthesis

The literature search initially identified 713 records across all databases. Following removal of duplicate records (*n* = 138), 575 records remained for title and abstract screening.

During the screening phase, 243 records were excluded due to lack of direct relevance to aMPV (*n* = 118), insufficient methodological detail (*n* = 39), conference-only publications (*n* = 24), inaccessible full texts (*n* = 18), or failure to meet eligibility criteria (*n* = 44).

Subsequently, 332 full-text articles were assessed for eligibility. Following full-text evaluation, 164 studies were excluded because they provided limited primary evidence (*n* = 56), represented duplicated datasets (*n* = 29), lacked sufficient epidemiological or molecular detail (*n* = 47), or did not align with the predefined objectives of the review (*n* = 32).

Ultimately, 168 studies were included in the final narrative synthesis.

The included studies were organized into thematic categories encompassing viral classification and genomic organization, host range and species susceptibility, epidemiology and geographic distribution, molecular evolution and subtype diversity, wildlife–poultry interactions, diagnosis and surveillance, vaccination, and control strategies.

Where overlapping findings were identified, greater interpretive emphasis was placed on recent molecular investigations, large-scale surveillance studies, longitudinal investigations, and publications providing comprehensive epidemiological, ecological, or phylogenetic characterization.

### 2.4. Quality Considerations

Given the broad scope of this narrative review and the heterogeneity of available evidence, a formal risk-of-bias scoring system or standardized quality assessment tool was not applied.

Nevertheless, methodological rigor was qualitatively considered during evidence synthesis. Particular attention was given to study design, sampling framework, diagnostic methodology, molecular characterization, epidemiological interpretation, and reporting transparency. Greater interpretive weight was assigned to studies providing robust molecular evidence, larger sample sizes, longitudinal surveillance, and comprehensive phylogenetic analyses.

### 2.5. Data Synthesis

Because substantial heterogeneity existed among study designs, host species, sampling frameworks, diagnostic approaches, geographic coverage, and surveillance intensity across countries and production systems, quantitative meta-analysis was not considered appropriate.

Instead, findings were synthesized narratively to facilitate critical comparison and integration of evidence related to epidemiological patterns, subtype distribution, ecological interactions, surveillance limitations, diagnostics, vaccination outcomes, and current knowledge gaps concerning the transmission ecology and molecular epidemiology of aMPV.

### 2.6. Methodological Limitations

This review was conducted as a narrative synthesis and therefore did not include formal study quality scoring, quantitative meta-analysis, or statistical pooling of outcomes.

Although this approach enabled integration of diverse evidence across epidemiology, molecular evolution, diagnostics, wildlife–poultry interactions, and disease control, it limits direct quantitative comparison between studies and does not permit estimation of pooled prevalence, effect sizes, or between-study variability.

Accordingly, the findings and conclusions presented should be interpreted as a qualitative synthesis intended to identify broad epidemiological patterns, highlight emerging trends and knowledge gaps, and support future research priorities rather than establish quantitative estimates of disease burden or causal relationships.

**Figure 1:**
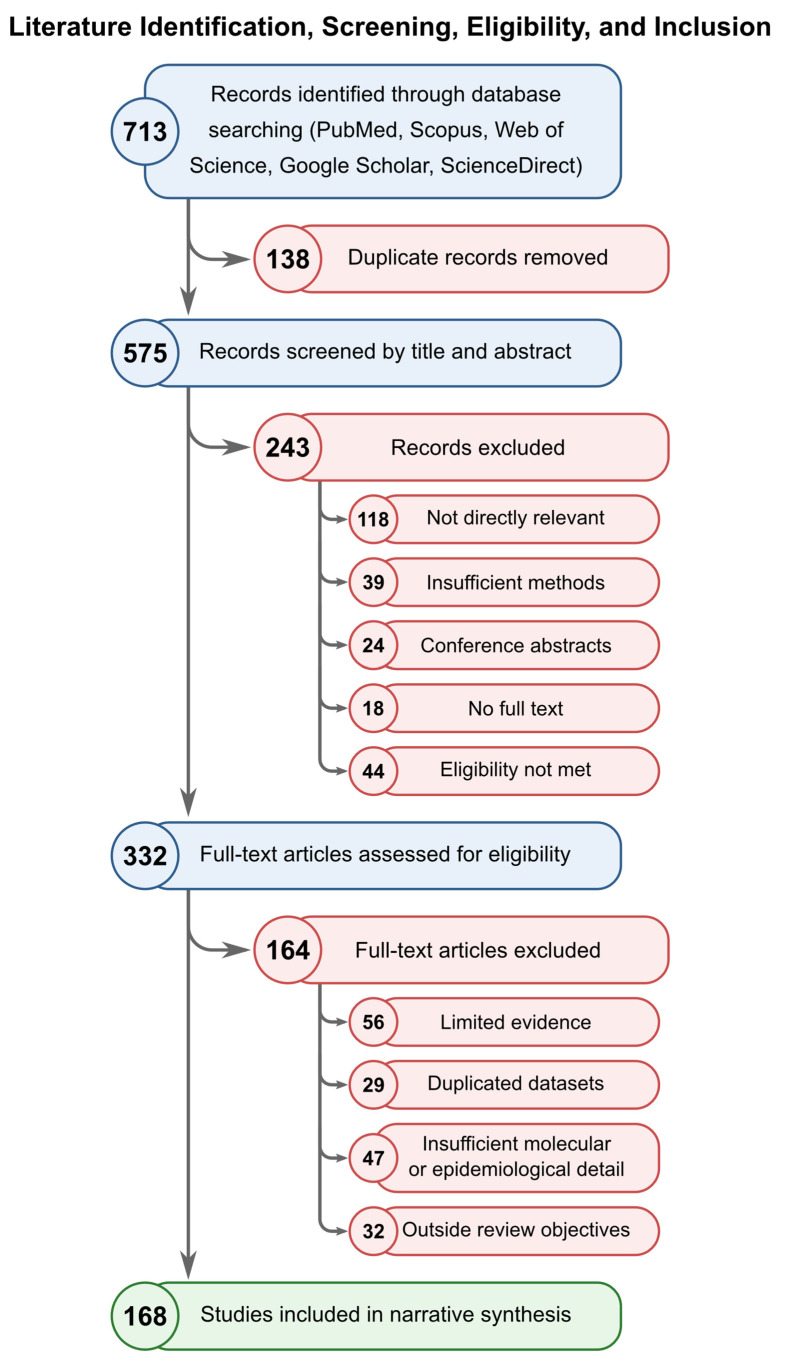
Flow diagram of literature identification, screening, eligibility assessment, and inclusion process. Blue: Investigated studies; Red: Excluded studies; Green: Final studies.

A study selection flow diagram summarizing identification, screening, eligibility assessment, exclusions, and final inclusion of studies is presented in Figure 1.

## 3. History, Classification and Characteristics of Avian Metapneumovirus

### 3.1. History and Origin of aMPV

aMPV was first identified in 1970 in South Africa during outbreaks of acute respiratory disease in turkeys, characterized by nasal discharge, conjunctivitis, infraorbital sinus swelling, respiratory distress, and mortality reaching up to 30%. This condition was subsequently designated TRT. The causative agent showed antigenic and biological relatedness to hRSV and murine pneumonia virus, supporting its classification as the first recognized avian pneumovirus [4,5,6,7].

Following its initial detection, aMPV was rapidly reported in turkey production systems in France and the United Kingdom, followed by dissemination across Western Europe, Asia, and South America [8,9,10]. Shortly thereafter, chickens in affected regions were associated with respiratory disease accompanied by facial edema, later described as SHS. Early attribution of SHS to bacterial pathogens such as *Escherichia coli* (*E.coli*) was later revised following serological detection of TRTV antibodies and isolation of TRTV-like viruses from chickens, confirming a common etiological agent for TRT and SHS. Since then, aMPV has been identified in broiler parent flocks across Europe, the United States, Africa, the Middle East, Asia, Brazil, and other regions globally [11,12].

### 3.2. Taxonomic Classification

aMPV was initially classified within the family *Paramyxoviridae*; however, advances in molecular virology and phylogenetic analysis led the International Committee on Taxonomy of Viruses in 2016 to the nascent family *Pneumoviridae* in the order *Mononegavirales*. This reclassification was based primarily on differences in genomic organization compared with *orthopneumoviruses*, including the absence of the nonstructural proteins NS1 and NS2, which are characteristic of several mammalian pneumoviruses such as human respiratory syncytial virus (hRSV) [13,14,15,16].

The family *Pneumoviridae* currently comprises two genera: *Orthopneumovirus* and *Metapneumovirus*. The genus *Orthopneumovirus* includes mammalian pneumoviruses such as *Orthopneumovirus hominis*, *Orthopneumovirus bovis*, and *Orthopneumovirus muris*, whereas the genus Metapneumovirus includes *Metapneumovirus avis* (avian metapneumovirus) and *Metapneumovirus hominis* (human metapneumovirus; hMPV) [1,2,3].

Based on genetic variability, antigenic characterization, virus neutralization assays, and ELISA cross-reactivity, aMPV is currently classified into four principal subgroups designated A, B, C, and D. Subgroups A, B, and D are genetically more closely related to each other, whereas subgroup C is more divergent and shares greater genomic similarity with hMPV in terms of nucleotide homology and genome organization. More recently, two genetically distinct aMPV-like viruses were identified in North American monk parakeets and great black-backed gulls, although their formal taxonomic classification has not yet been established. Phylogenetic analyses suggest that avian and human metapneumoviruses may share an ancestral evolutionary origin, potentially linked to bat-associated metapneumoviruses [17,18].

### 3.3. Morphology and Genomic Organization

aMPV manifests as an enveloped, negative-sense, non-segmented, single-stranded RNA virus, harboring a genome of 13.2–15.3 kb. Virions display pleomorphism, assuming spherical or filamentous conformations with diameters of 80–200 nm, extensible to 600 nm in filamentous variants. The envelope, derived from host plasma membrane, is buttressed by an internal matrix protein stratum encasing the helical nucleocapsid [2,16].

**Figure 2:**
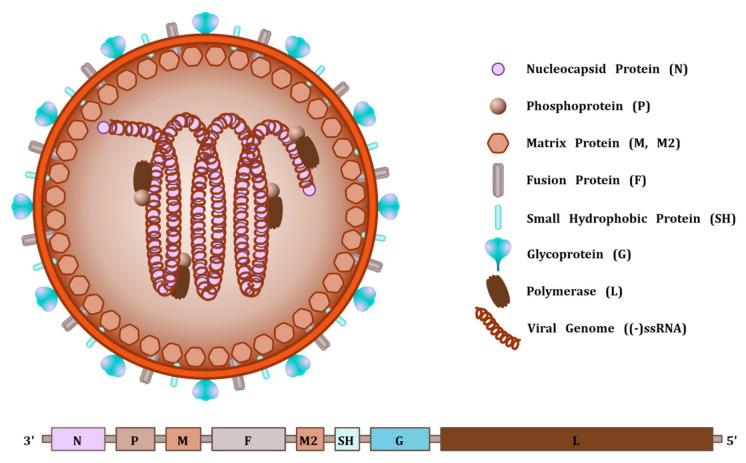
Structural and genomic organization of avian metapneumovirus (aMPV), showing the enveloped virion structure and the principal viral proteins encoded by the negative-sense single-stranded RNA genome, including N, P, M, F, M2, SH, G, and L proteins involved in viral replication, attachment, fusion, and pathogenicity.

The genomic architecture adheres to the invariant order 3′-leader–N–P–M–F–M2–SH–G–L–trailer–5′, transcribing nine proteins from eight genes, wherein the M2 gene bears overlapping open reading frames (M2-1, M2-2). These comprise nucleoprotein (N), phosphoprotein (P), matrix (M), fusion (F), small hydrophobic (SH), attachment glycoprotein (G), and large RNA-dependent RNA polymerase (L). As a negative-sense RNA entity, genomic infectivity mandates ribonucleoprotein (RNP) complexation with N, P, and L to inaugurate transcription and replication. The helical nucleocapsid is enveloped by matrix protein and a lipid bilayer bearing glycoproteins F, SH, and G, pivotal to entry, morphogenesis, and immune subterfuge [16,19,20,21,22]. The structural and genomic organization of aMPV is illustrated in Figure 2.

The nucleoprotein (N; ~391 aa) encapsidates the viral RNA genome and represents one of the most conserved viral proteins. The phosphoprotein (P; ~279 aa) functions as an essential cofactor for the large RNA-dependent RNA polymerase (L; ~2004 aa), and together these proteins mediate transcription and replication of the viral genome. The matrix protein (M) plays a central role in virion assembly and viral egress, whereas the M2 gene encodes two overlapping proteins: M2-1, which enhances transcriptional processivity and read-through, and M2-2, which regulates the balance between transcription and genome replication [20].

Among the membrane-associated proteins, the attachment glycoprotein (G) and fusion protein (F) are the principal determinants of viral infectivity, pathogenicity, and immunogenicity. The G protein is a highly glycosylated type II membrane protein (approximately 389–585 aa), enriched in serine, threonine, and proline residues, and exhibits considerable genetic variability among aMPV subgroups. It contains major neutralizing epitopes and serves as the primary molecular marker for subtype classification and molecular epidemiological investigations. Notably, the G protein of subtype C is longer than those of other aMPV subgroups [23,24,25].

In contrast, the F protein (≈538 aa) is relatively conserved across subgroups and is synthesized as an inactive precursor (F0), which is cleaved by host serine proteases, including TMPRSS12, into the active F1 and F2 subunits. This cleavage enables pH-independent membrane fusion and syncytium formation through mediation of fusion between the viral envelope and host cell membrane. Although membrane fusion is generally independent of the G protein, trypsin supplementation can enhance viral entry of subtype C—but not subtypes A or B—possibly due to differences in fusogenic residues [26,27].

The small hydrophobic (SH) protein (≈180 aa) is a type II integral membrane protein that occurs in both glycosylated and non-glycosylated forms and functions as a viroporin. SH contributes to modulation of host cell membrane permeability and host immune responses, further influencing viral pathogenesis [28,29,30].

### 3.4. Physical and Chemical Characteristics

As an enveloped virus, aMPV evinces exquisite sensitivity to thermal extremes, pH fluctuations, lipid solvents, and standard disinfectants. Viability endures ~12 weeks at 4 °C, >24 weeks at −20 °C, yet dissipates within days at 37 °C. In poultry litter, pathogenicity persists 60 days at −12 °C, with RNA detectable >90 days at 8 °C. Susceptibility to sodium hypochlorite, alcohols, quaternary ammoniums, phenolics, and iodophors swiftly abrogates infectivity. Stability is confined to pH 5–9, with prompt inactivation extraneous thereto. These traits profoundly modulate persistence, transmission, and biosecurity efficacy in poultry enterprises [31,32,33].

## 4. Transmission Ecology and Epidemiology of aMPV

### 4.1. Host Range and Species Susceptibility of aMPV

aMPV infects a broad range of avian hosts, including domestic poultry, wild and migratory birds, and several game and non-gallinaceous species. Variations in susceptibility, clinical presentation, and epidemiological patterns are influenced by host species, age, production system, and viral subtype, collectively shaping the ecological dynamics of the virus [34].

Among domestic poultry, turkeys are considered the principal natural host and represent the species in which aMPV was first identified. Infection is classically associated with TRT, the most severe clinical manifestation of aMPV infection. Disease occurs most frequently in poults between 3 and 12 weeks of age, particularly during the period of highest susceptibility at approximately 4–9 weeks. Affected flocks may exhibit respiratory distress, infraorbital sinus swelling, nasal and ocular discharge, reduced weight gain, and significant economic losses. Turkeys are also capable of transmitting all major aMPV subtypes, although subtype-specific variation exists in clinical severity and tissue tropism, contributing to differences in outbreak dynamics across production systems [4].

Chickens are also susceptible to aMPV infection, although clinical disease is generally more variable and often less severe than in turkeys. Infection has been documented across different production categories and age groups; however, respiratory disease is more commonly recognized in broilers because of intensive husbandry practices, high stocking density, and shorter production cycles. Clinical outbreaks frequently occur following decline of maternally derived antibodies, with susceptibility increasing after approximately 3–4 weeks of age. In breeder and layer flocks, aMPV infection has been associated with SHS, reductions in egg production, impaired shell quality, and increased susceptibility to secondary bacterial infections. Importantly, infected chickens may seroconvert despite limited or transient viral shedding, potentially complicating diagnosis and facilitating unnoticed field circulation. This seroconversion–shedding dissociation is particularly relevant in field surveillance, where serological positivity may not correlate with active viral detection, leading to underestimation of infection prevalence in chicken populations [4,5,6,7,35].

Beyond gallinaceous poultry, aMPV has been detected in numerous domestic and game bird species including ducks, geese, pigeons, pheasants, quail, guinea fowl, and ostriches. In many of these hosts, infection appears subclinical or mild under field conditions. Nevertheless, their possible role in viral maintenance, environmental dissemination, or interspecies transmission remains under investigation. Notably, infection in pheasants, guinea fowl, and ostriches highlights that aMPV is not restricted to conventional poultry species, but can extend into game birds and ratites, although often with predominantly serological rather than clinical evidence [36,37,38,39,40,41].

Wild and migratory birds have increasingly attracted attention because of their potential involvement in aMPV ecology and long-distance dissemination. Molecular and serological evidence of infection has been reported in ducks, geese, gulls, sparrows, swallows, starlings, pigeons, and other water-associated avian species, with most detections occurring in apparently healthy birds. These findings suggest possible ecological connectivity between wildlife and poultry populations, particularly along migratory flyways and shared aquatic habitats. However, definitive evidence regarding long-term reservoir competence, viral persistence, and transmission directionality remains limited. Importantly, wild birds often demonstrate transient or low-level viral detection with relatively higher seroprevalence, suggesting intermittent exposure rather than sustained replication in many species [42].

Subtype-specific host associations further contribute to the epidemiology of aMPV. Subgroups A and B are mostly detected in chickens and turkeys and exhibit substantial antigenic and genetic similarity. Subtype D, originally identified in France, is genetically distinct from subtypes A–C and has demonstrated experimental infectivity in galliform birds. In contrast, subtype C displays greater ecological diversity, with Eurasian strains commonly associated with ducks and North American strains more frequently linked to turkeys.

Notably, subtype C predominates among many wild bird detections, whereas reports of subtypes A and B in wildlife remain comparatively infrequent. Subtype C is increasingly recognized as a cross-species bridging lineage, capable of infecting both waterfowl and gallinaceous hosts, often resulting in seroconversion without consistent viral shedding in chickens. More recently, genetically divergent aMPV-like viruses were identified in monk parakeets and great black-backed gulls. These viruses occupy intermediate phylogenetic positions between classical aMPVs and human metapneumoviruses, suggesting that the evolutionary diversity and host range of avian metapneumoviruses may be broader than currently recognized [8,42].

Moreover, important gaps remain regarding the epidemiological significance of many non-gallinaceous hosts and the long-term ecological role of wildlife in viral maintenance and dissemination. Continued integrated surveillance combining poultry, wildlife, and molecular epidemiology is therefore necessary to better understand the transmission ecology and evolutionary dynamics of aMPV across different geographic regions and production systems [4,5,41,42].

### 4.2. Global Emergence of aMPV

The global dissemination of aMPV has been closely linked to the intensification and expansion of commercial poultry production. Following its establishment in Europe during the mid-1980s, the virus was subsequently reported in Italy, Germany, Spain, Hungary, and additional countries. Phylodynamic analyses suggest introduction into Europe around this period, followed by rapid spread across Western and Mediterranean regions during the 1990s and subsequent establishment in Eastern Europe and other continents. Molecular characterization during this period identified two major subgroups, A and B. Although early observations suggested partial geographic clustering, subsequent evidence demonstrated co-circulation of both subgroups across poultry-producing regions. Over time, subtype B became the predominant lineage in many countries, potentially reflecting improved transmission dynamics and adaptation under intensive production conditions [40,41,42,43,44].

Subtype C was later recognized as a genetically distinct lineage comprising North American and Eurasian clades. The North American lineage was first identified in the United States during the mid-1990s, whereas Eurasian strains were subsequently reported in Europe and China. In contrast, subtype D, first identified in France around 2000, has remained geographically restricted with limited epidemiological distribution [39,40,41,42].

Historically, subtype distribution demonstrated regional structuring, with subtypes A and B predominantly reported in Europe and the United Kingdom, subtype C in North America, and subtype D confined to France [39,40,41,42,43,44,45,46]. However, increasing poultry trade, production intensification, and interactions at domestic–wild bird interfaces have progressively reduced these geographic boundaries.

Recent epidemiological evidence indicates renewed global expansion of aMPV. Subtypes A and B have re-emerged in the United States after prolonged absence since the early 2000s. Phylogenetic analysis of recent subtype A isolates from California demonstrated close genetic relatedness to strains previously reported in Mexico, suggesting possible transboundary spread. Subtype B has continued to expand geographically and has recently been reported in Iraq (2022), Brazil and Tunisia (2023), and Morocco (2024), where widespread detection in broiler systems suggests established circulation. Increasing detection of subtype C in both domestic and wild birds further supports its ecological adaptability and potential for cross-species transmission. In Italy, subtype C was identified in wild birds, while reports from China confirmed circulation in domestic waterfowl [1,2,6,40,41,42,43,44,45,46].

Although wild birds are increasingly implicated in aMPV ecology, particularly for subtype C, current evidence is largely based on molecular detection; therefore, their reservoir competence and contribution to long-term viral maintenance remain uncertain.

More recently, genetically divergent aMPV-like viruses have been identified in wild birds, including North American aquatic species and monk parakeets. These viruses exhibit only approximately 61–66% genomic similarity to classical subtypes A–D, indicating substantial evolutionary divergence and supporting the emergence of novel genetic lineages within the genus Metapneumovirus [47,48,49].

Additional novel strains identified in gull species, including herring gulls and great black-backed gulls, form intermediate phylogenetic clusters between classical avian and mammalian metapneumoviruses, suggesting that aMPV evolutionary diversity may be broader and more complex than previously recognized [41,42,43].

**Figure 3:**
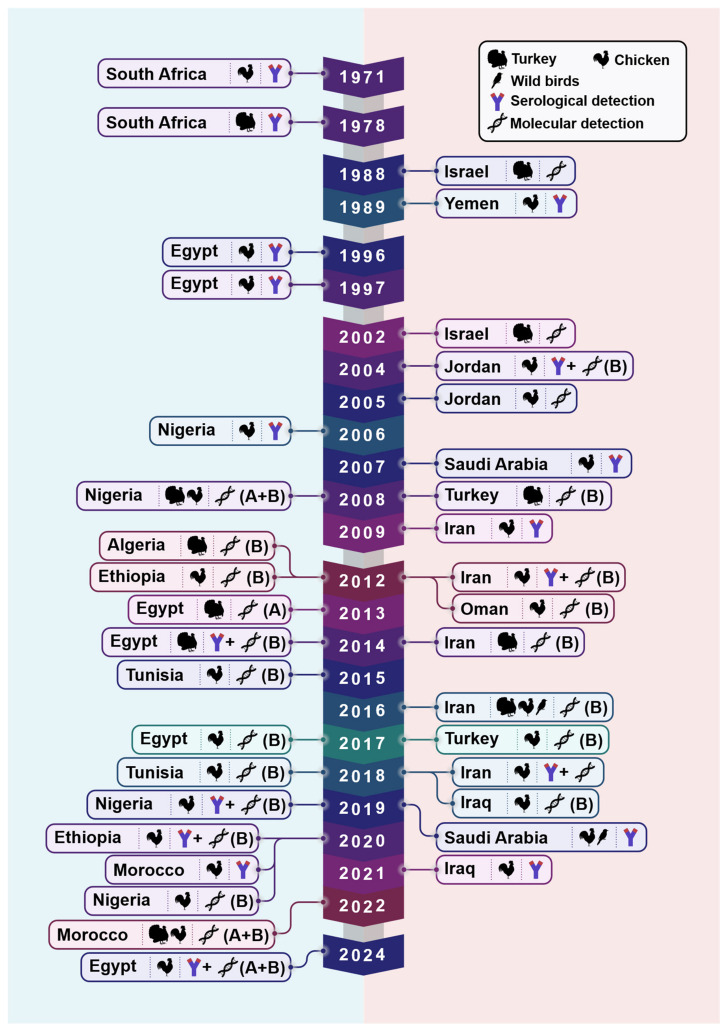
Chronological timeline summarizing published reports of serological and molecular detection of avian metapneumovirus (aMPV) in poultry and wild birds across Africa and the Middle East from 1971 to 2024. The figure presents the reported host species, geographic distribution, diagnostic approaches, and identified subtypes (A and/or B) according to available epidemiological investigations. Serological evidence is represented by ELISA-based detection, whereas molecular identification includes RT-PCR and sequencing-based approaches.

The global epidemiology of aMPV is characterized by progressive geographic expansion, subtype replacement, and increasing detection of genetically divergent strains. While subtype B remains the most widely distributed lineage in poultry, emerging evidence highlights ongoing viral evolution and the appearance of novel genetic groups. These findings emphasize the dynamic nature of aMPV ecology and the importance of continued molecular surveillance across poultry and wild bird populations. Major historical developments in aMPV research are summarized in Figure 3.

### 4.3. Transmission Pathways and Drivers of aMPV Spread

aMPV is primarily transmitted through direct contact between infected and susceptible birds and represents the principal route sustaining viral spread. Infection typically occurs following exposure to aerosolized particles or respiratory secretions released by infected birds, particularly under close-contact conditions in confined housing systems. Viral shedding is largely limited to the early phase of infection, indicating the absence of a latent or chronic carrier state and contributing to relatively limited environmental persistence. Nevertheless, this short shedding period remains sufficient to sustain efficient flock-level transmission [48].

Transmission efficiency is further enhanced by modern poultry production practices. High stocking densities, shared airspace, and synchronized production cycles facilitate rapid amplification of infection. Chickens have been identified as efficient sources of infection and implicated in transmission to neighboring turkey flocks, emphasizing the vulnerability of multi-species production systems and densely populated poultry regions [44,48].

Although no biological or mechanical arthropod vectors have been linked to aMPV spread, indirect transmission remains epidemiologically relevant. Contaminated litter, bedding, transport vehicles, equipment, personnel, and other fomites may contribute to dissemination between flocks, particularly where biosecurity implementation is inconsistent [49].

Despite the recognized reproductive tract tropism of aMPV, available evidence does not support vertical transmission as an epidemiologically significant pathway. Viral RNA has been detected in duck semen, cockerel testes from farms with fertility disorders, and eggs from specific pathogen-free turkeys experimentally infected with subtype C; however, these findings are generally considered incidental rather than evidence of efficient vertical spread. Current evidence indicates that horizontal transmission remains the dominant mechanism underlying viral maintenance and dissemination [50,51,52].

At a broader scale, structural and ecological factors facilitate aMPV dissemination. Intensification of poultry production, expansion of live bird and hatching egg trade, and increasing integration of production systems promote long-distance viral movement. Concurrently, gaps in biosecurity implementation, particularly in rapidly developing poultry sectors, support continued circulation and repeated introduction events. Together, these drivers reinforce transmission and contribute to endemic virus persistence in many production settings [53,54].

### 4.4. Wild Birds as Drivers of aMPV Ecology and Inter-Species Transmission

Interest in the role of wild birds in aMPV ecology has increased alongside the expanding geographic distribution of the virus and growing interactions between wildlife and poultry systems. Similar to avian influenza viruses (AIVs) and Newcastle disease virus (NDV), wildlife–poultry interfaces are increasingly recognized as potential points of viral introduction and dissemination. Wild birds are therefore considered potential contributors to aMPV maintenance and spread, particularly subtype C, although evidence for circulation of additional subtypes remains limited but increasing [55,56].

Early molecular and serological investigations demonstrated susceptibility or exposure to aMPV across multiple wild bird taxa, including ducks, geese, gulls, sparrows, swallows, pigeons, and shorebirds across Europe, North America, South America, and Asia [1,2,3]. These observations suggest that the ecological host range of aMPV extends beyond commercial poultry and may involve avian communities occupying wetlands, migratory stopovers, and peri-domestic habitats [57,58].

Subtype C remains the subtype most consistently identified in wild birds. Studies from North America and Europe demonstrated genetic relatedness between subtype C strains detected in wild waterfowl and those recovered from domestic turkeys and chickens, supporting possible cross-species transmission. In the United States, subtype C has been predominantly associated with turkeys, whereas in Europe it has more frequently been identified in ducks and migratory waterfowl, indicating regional variation in host ecology [56,59,60].

Yao et al. reported isolation and molecular characterization of subtype C from chickens in Jiangsu Province, China, with phylogenetic clustering alongside waterfowl-associated strains, supporting potential spillover between aquatic birds and poultry [61]. Likewise, Sun et al. isolated subtype C from Muscovy ducks, indicating circulation within domestic and semi-domestic waterfowl populations [62].

European surveillance studies have also documented aMPV detection in wild birds. Tucciarone et al. identified subtypes A, B, and C among migratory and resident birds in northern-central Italy [63]. Although these findings demonstrate genetic diversity, their epidemiological implications remain uncertain.

Lupini et al. reported subtype B detection in a Northern shoveler (Spatula clypeata) wintering in Italy, suggesting that poultry-associated lineages may also occur in migratory birds [64]. However, transmission directionality could not be determined.

Studies from South America similarly support interactions between wildlife and poultry systems. Felippe et al. identified subtypes A and B in feral pigeons and wild birds in Brazil and reported genetic similarity to poultry strains [65]. Rizotto et al. described re-emergence of subtype A in wild birds in São Paulo State following apparent disappearance from commercial poultry, while Escobar-Alfonso et al. documented subtype B circulation in both poultry and wild birds in Colombia [66,67].

Experimental studies further support host susceptibility. Cha et al. demonstrated that subtype C isolates originating from wild birds replicated in domestic turkeys, producing respiratory disease and viral shedding under controlled conditions [68]. However, these findings do not establish the frequency of such events under field conditions.

Recent evidence from Egypt further highlights the relevance of wildlife–poultry interactions. Molecular and serological investigations detected exposure and occasional identification of subtype B in wild birds from migratory and poultry-dense regions. Higher seropositivity relative to RT-PCR positivity suggests previous exposure rather than active infection. Combined with subtype B detection in unvaccinated broiler breeder flocks, these findings indicate possible epidemiological connectivity among wild birds, backyard poultry, and commercial systems, although direct transmission pathways remain unresolved [69,70].

Egypt represents a particularly relevant setting for aMPV ecology because of its location along major migratory flyways connecting Europe, Asia, and Africa. Wetland ecosystems in the Nile Delta and Mediterranean coast, including Lake Manzala, Lake Burullus, and the Suez Canal region, create opportunities for contact between migratory birds and poultry production areas, potentially facilitating indirect exposure where biosecurity is limited [71,72,73].

Despite increasing evidence of wildlife involvement, substantial knowledge gaps remain regarding reservoir competence, transmission frequency, and mechanisms sustaining viral persistence. Integrated surveillance incorporating wildlife monitoring, poultry surveillance, and phylogenetic analysis is therefore needed to improve understanding of aMPV epidemiology and strengthen risk assessment and control strategies.

### 4.5. Epidemiology of aMPV in Africa

**Figure 4:**
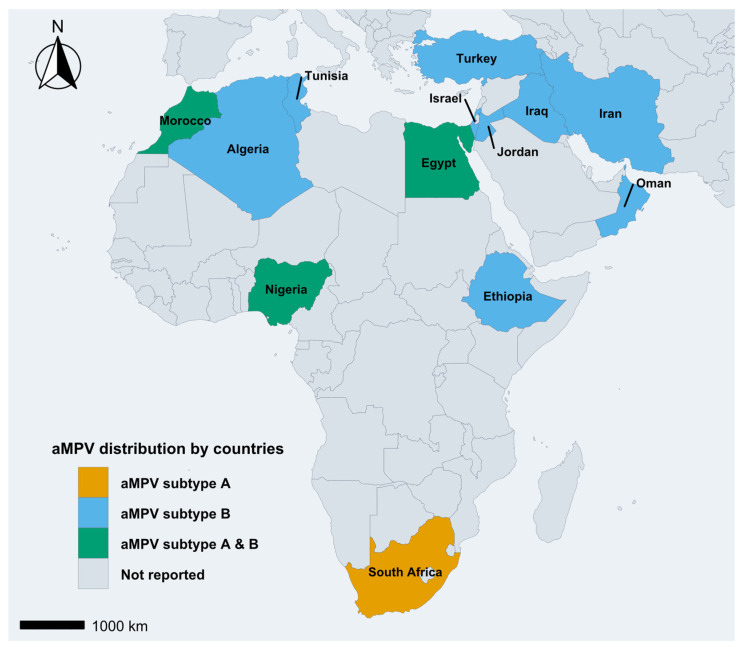
Geographic distribution of avian metapneumovirus (aMPV) subtypes reported in Africa and the Middle East countries based on available published epidemiological data. Countries are categorized according to the reported detection of subtype A, subtype B, co-circulation of subtypes A and B, or absence of available reports, illustrating the current regional distribution patterns of circulating aMPV subtypes.

aMPV is increasingly recognized as an endemic and economically important respiratory pathogen in several African poultry production systems. Although extensively characterized in Europe, Asia, and the Americas, its epidemiology in Africa has historically remained less defined due to limited diagnostic capacity, underreporting, and the frequent masking of infections within complex respiratory disease syndromes involving multiple viral and bacterial co-infections. Over the past two decades, accumulating serological and molecular evidence has confirmed that aMPV is widely distributed across North, West, and East Africa, with subtype B predominating in most regions and subtype A detected sporadically. The observed epidemiological patterns likely reflect a combination of poultry trade movements, vaccine-associated strain circulation, local production system characteristics, and ecological interactions, including possible involvement of migratory wild birds [1,2,13,25,42]. The global distribution and subtype diversity of aMPV are summarized in Figure 4.

In North Africa, aMPV has been retrospectively detected in Tunisia in respiratory disease cases collected between 2015 and 2019, where subtype B was the only lineage identified. Phylogenetic analysis suggests delayed recognition of previously circulating viruses and possible multiple introduction events or strain replacement linked to poultry movement and trade [74,75]. In Algeria, subtype B has been detected mainly in association with respiratory disease outbreaks involving multiple viral and bacterial pathogens, including Infectious bronchitis virus (IBV), AIV, and Mycoplasma spp., indicating that aMPV contributes primarily as part of polymicrobial respiratory disease complexes rather than as a primary pathogen [76]. In Morocco, both serological and molecular data confirm endemic circulation of subtype B, with high seroprevalence in unvaccinated flocks. Detection of subtypes A and B in wild birds further indicates ecological overlap between poultry and migratory avifauna, suggesting a role for Morocco as an interface for Afro–Eurasian viral exchange along migratory flyways [77,78,79].

In West Africa, Nigeria provides evidence of long-term endemic circulation with detection of both subtype A and subtype B in poultry populations. Serological studies indicate high exposure levels in unvaccinated flocks, while molecular data confirm subtype B predominance and frequent co-infections with bacterial respiratory pathogens such as Escherichia coli and Pseudomonas aeruginosa. These findings support sustained viral circulation within multi-pathogen respiratory disease systems influenced by climatic variability and production conditions [80,81,82].

In East Africa, evidence remains limited and fragmented. In Ethiopia, subtype B was detected in breeder flocks in association with other respiratory pathogens, while subsequent surveillance failed to detect the virus in sampled farms. This inconsistency likely reflects limited sampling coverage and non-continuous surveillance rather than true absence of infection, indicating the need for systematic longitudinal studies to clarify epidemiological status [83,84].

Egypt represents a partially characterized but increasingly important setting for aMPV epidemiology in Africa. Although available studies remain fragmented and geographically uneven, Egypt has substantial epidemiological relevance owing to its extensive poultry industry, heterogeneous biosecurity implementation, and strategic location along major migratory bird flyways. The country hosts one of the largest and most densely interconnected poultry production sectors in the Middle East and North Africa, encompassing intensive commercial enterprises, semi-closed production systems, live bird markets, and extensive backyard poultry holdings that frequently coexist within the same geographic areas. Commercial broiler, breeder, layer, turkey, and duck production is concentrated primarily in the Nile Delta and Canal governorates, including Dakahlia, Sharqia, Kafr El Sheikh, Beheira, Gharbia, Ismailia, Port Said, and Damietta governorates [69,70].

These high-density production areas overlap with ecologically important environments, including wetlands, irrigation networks, fish farming regions, and major migratory bird habitats such as Lake Manzala, Lake Burullus, and the Suez Canal corridor, creating favorable conditions for viral introduction, maintenance, and local dissemination. Beyond intensive production zones, rural poultry production remains an integral component of agricultural systems throughout the Nile Valley and Middle Egyptian governorates, including Fayoum, Beni Suef, Minya, Assiut, and Sohag. Backyard systems commonly operate under low-biosecurity conditions where chickens, ducks, geese, pigeons, and turkeys are reared in close proximity to human settlements, irrigation canals, agricultural land, and free-ranging synanthropic birds. Informal poultry holdings are frequently situated near drainage canals, fishponds, and peri-wetland environments, increasing opportunities for indirect contact between domestic poultry and migratory waterfowl [70,71].

Early epidemiological evidence suggested that aMPV had circulated in Egyptian poultry populations long before molecular confirmation became available. Initial serological investigations during the 1990s demonstrated widespread antibody exposure associated with SHS and reproductive performance deterioration in commercial poultry, indicating silent endemic circulation and historical viral presence [85,86]. Additional evidence from commercial turkey production was provided by Mahmoud et al. (2008), who investigated TRT/aMPV in Egyptian commercial turkey flocks and demonstrated extensive field exposure, reporting an overall ELISA seropositivity of 87.5% (140/160), while RT-PCR confirmed active viral detection in field samples, supporting sustained circulation of aMPV in both clinically affected and apparently healthy flocks [87].

The transition from serological evidence to molecular confirmation occurred in 2014 when subtype A was identified in clinically affected turkey poults in Fayoum, representing the first molecularly confirmed report of aMPV infection in Egypt and establishing active field circulation under commercial production conditions [88]. Subsequent molecular investigations identified subtype B as the predominant lineage circulating across multiple governorates, including Cairo, Giza, and Beni Suef. Several isolates exhibited close genetic relatedness to vaccine-derived strains, raising concerns regarding vaccine-associated viral evolution, field persistence of vaccine-like variants, or shared ancestry between circulating and vaccine strains [89].

Field investigations further established the clinical significance of aMPV within respiratory disease complexes in Egyptian poultry. Multiple studies linked aMPV infection to swollen head syndrome and respiratory disease outbreaks frequently accompanied by opportunistic bacterial pathogens, particularly Escherichia coli and Pseudomonas aeruginosa, supporting the role of aMPV as an initiator of epithelial damage that predisposes birds to secondary bacterial colonization and increased disease severity [90,91,92].

Serological surveillance across different production sectors subsequently demonstrated broad host exposure extending beyond turkeys to include broilers, layers, and ducks, indicating widespread endemic circulation across poultry populations [93]. Large-scale surveillance of broiler breeder flocks later revealed extremely high seroprevalence levels approaching complete flock exposure, suggesting sustained viral maintenance, repeated transmission cycles, and long-term endemicity. Concurrent molecular screening consistently identified subtype B as the dominant circulating genotype throughout production cycles [69,94].

More recently, integrated molecular and serological investigations expanded the epidemiological framework of aMPV in Egypt beyond domestic poultry. Subtype B was identified in multiple wild bird species, including Columba livia, Anas platyrhynchos, Bubulcus ibis, and Corvus cornix, with phylogenetic analyses demonstrating close molecular relationships between poultry- and wildlife-derived strains. These findings support ecological connectivity between poultry production systems and surrounding ecosystems and suggest that wild birds may contribute to environmental maintenance and dissemination of aMPV in regions characterized by high poultry density and overlapping migratory flyways [69,94].

Egypt’s geographic position along major migratory routes, together with intensive poultry production concentrated in the Nile Delta and surrounding governorates, may facilitate repeated viral introduction and long-term persistence. Wetlands including Lake Manzala and Lake Burullus likely function as ecological interfaces supporting indirect transmission through shared environments and contaminated resources linking wild birds, backyard poultry, and commercial operations [73,93,94].

Overall, the Egyptian epidemiological trajectory illustrates a progressive transition from early serological detection to molecular confirmation, subtype diversification, endemic breeder circulation, and ultimately recognition of wildlife-associated ecological maintenance. The predominance of subtype B, persistent circulation across production systems, frequent involvement in mixed respiratory disease complexes, and emerging evidence of wildlife–poultry connectivity collectively indicate that aMPV ecology in Egypt is multifactorial and dynamic. These observations highlight the need for integrated One Health surveillance frameworks that combine systematic monitoring of commercial poultry, backyard sectors, and wildlife reservoirs to better understand transmission dynamics and inform long-term control strategies.

### 4.6. Epidemiology of aMPV in the Middle East

The Middle East represents an important epidemiological interface for aMPV due to intensive poultry production, active regional trade, high farm density, and its strategic location along major Afro–Eurasian migratory bird flyways. Since the early 2000s, multiple countries have documented aMPV circulation, with subtype B emerging as the predominant lineage, whereas subtype A has been reported less frequently. Current evidence supports the establishment of aMPV in both commercial and backyard poultry systems, where it commonly contributes to multifactorial respiratory disease under variable biosecurity conditions [95].

In Iraq, aMPV was first molecularly confirmed between September 2018 and August 2019 in broiler flocks affected by SHS. Al-Hasan et al. (2022) detected aMPV in 23.8% of 67 commercial farms, with all sequences classified as subtype B and no evidence of subtypes A or C, confirming the association of aMPV-B with SHS outbreaks [96]. Subsequent investigations demonstrated frequent involvement of aMPV-B in mixed respiratory disease complexes, particularly with ORT, M.S, M.G, and variant infectious bronchitis virus (793B genotype), detected in 41.7% of examined samples, including multidrug-resistant isolates. Serological data from Duhok Province further identified antibodies in 26.6% of sera and 40% of flocks despite the absence of vaccination, supporting persistent subclinical transmission [97].

Iran provides one of the most comprehensive epidemiological datasets in the region. The first molecular detection was reported in 2012 in broilers, where subtype B showed phylogenetic similarity to strains from Africa, Asia, and South America, suggesting broad lineage connectivity [98]. Subsequent studies confirmed subtype B in turkeys (4.1%) [99] and documented widespread detection in broiler flocks across multiple provinces, with prevalence ranging from 23% to 65% among birds with respiratory disease [100,101,102,103]. Live bird markets were identified as important epidemiological nodes, with 30.6% positivity across chickens, turkeys, and wild birds, all classified as subtype B [104]. Serological investigations additionally demonstrated high exposure rates reaching 48.1% in broilers and 93.2% in breeders, indicating extensive transmission across commercial, backyard, market, and wildlife-associated systems [105].

In Oman, surveillance conducted between June and September 2012 identified low-level aMPV circulation in backyard poultry. Al-Shekaili et al. detected subtype B in 2.06% of unvaccinated flocks sampled across all governorates. Positive birds were clinically healthy and restricted to chickens, suggesting subclinical infection and possible involvement of backyard populations in local virus maintenance [106].

In Turkey, molecular investigations confirmed aMPV occurrence in commercial poultry. Bayraktar et al. (2018) detected subtype B in 7.2% of broiler flocks and reported phylogenetic similarity to Israeli and vaccine-associated strains, suggesting regional movement or vaccine-related detection [107]. Ardıçlı et al. (2022) later identified the virus in broilers (8.33%) and layers (13.33%), frequently in association with infectious bronchitis virus and avian reovirus, emphasizing the contribution of aMPV to multifactorial respiratory disease [108].

Israel represents one of the earliest and most extensively investigated settings for aMPV in the region. Between 2002 and 2004, Banet-Noach et al. (2005) identified infection in 44% of examined flocks and detected both subtypes A and B, although subtype B predominated. Detection of field strains in vaccinated flocks suggested incomplete protection or circulation of heterologous variants [109]. Longitudinal studies subsequently demonstrated repeated recovery of field viruses in vaccinated turkey and chicken populations, supporting continued transmission in high-density production systems and highlighting the importance of molecular surveillance [110].

In Saudi Arabia, early serological investigations demonstrated exposure to aMPV in commercial poultry, with antibodies detected in 9.2% of broilers, predominantly among older birds. More recent studies reported 20.1% seropositivity in unvaccinated backyard chickens, supporting continued circulation, whereas wild Columbidae species tested negative under the investigated conditions [111,112].

In Jordan, Gharaibeh and Algharaibeh (2007) reported early serological and molecular evidence of aMPV, with antibodies detected in broilers (21.7%), layers (75%), and breeders (100%), alongside molecular confirmation of subtype B. Roussan et al. (2008) further supported the involvement of aMPV in multifactorial respiratory syndromes affecting poultry production [113,114].

Evidence from Yemen remains scarce. A single historical report described SHS in poultry (Sarakbi, 1989), without subsequent molecular confirmation or subtype characterization [115]. This highlights a major surveillance gap, and although circulation remains unconfirmed, its presence cannot be excluded within the broader regional poultry network.

Overall, the epidemiology of aMPV in the Middle East is characterized by widespread but heterogeneously documented circulation, with subtype B predominating across most investigated settings. Transmission appears to involve interconnected commercial production, backyard flocks, and live bird markets, with additional contributions from regional trade and production intensity. Despite differences in surveillance capacity, available evidence supports the endemic establishment of aMPV across multiple parts of the region. Overall, available evidence indicates that aMPV is widely established across poultry-producing regions of Africa and the Middle East, with subtype B representing the predominant lineage and subtype A reported less frequently (Table 1).

## 5. Diagnosis of aMPV Infection

Accurate diagnosis of aMPV infection is essential for disease control, surveillance, epidemiological investigations, and evaluation of vaccination programs. Diagnosis remains challenging because clinical manifestations are non-specific, viral shedding is short-lived, co-infections are common, and substantial genetic diversity exists among circulating subtypes. Disease expression may further vary according to bird age, immune status, environmental conditions, vaccination history, and concurrent infections. Consequently, laboratory confirmation using complementary direct and indirect diagnostic methods is required, as clinical assessment alone is insufficient [116,117,118,119].

Clinically, infected birds commonly present with nasal discharge, sneezing, coughing, conjunctivitis, infraorbital sinus swelling, facial edema, and tracheal rales. In breeders and layers, reproductive involvement may manifest as transient declines in egg production and eggshell quality, reduced fertility, poor hatchability, and decreased chick quality, contributing substantially to production losses [120,121,122]. However, these findings are not specific and overlap with those associated with other respiratory pathogens including AIV, NDV, IBV, *Mycoplasma gallisepticum* and *Mycoplasma synovie*.

Gross lesions are generally restricted to the upper respiratory tract and include catarrhal rhinitis, sinusitis, tracheitis, and airsacculitis. Histopathological changes commonly include epithelial degeneration, deciliation, mucosal hyperplasia, inflammatory infiltration, and ciliostasis. Although pathological examination is rapid and inexpensive, its diagnostic specificity remains limited because lesions are not pathognomonic and may be altered by secondary bacterial infections, particularly *Escherichia coli* [118,119].

Diagnostic performance depends strongly on appropriate sampling strategy and timing. Viral replication reaches peak levels during the early stage of infection, typically within the first 3–5 days after onset of clinical signs, after which viral loads decline rapidly due to local immune responses and epithelial clearance. Delayed specimen collection is therefore a major contributor to false-negative molecular and virological results. Recommended samples include choanal cleft, oropharyngeal and tracheal swabs, as well as respiratory tissues; investigations in breeders may additionally include reproductive tissues due to evidence of extra-respiratory involvement [118,123]. Sampling errors, insufficient epithelial collection, pooling practices, and cold-chain disruption may further reduce diagnostic sensitivity.

Virus isolation remains valuable for antigenic characterization and pathogenesis research. Tracheal organ cultures (TOC) provide functional assessment through demonstration of ciliostasis and epithelial injury following infection [124,125,126]. Embryonated eggs and cell culture systems may also be used, although repeated passages are often necessary before detectable replication or cytopathic effects occur. However, routine application is constrained by viral fragility, specialized requirements, and prolonged turnaround time.

Molecular methods currently represent the primary approach for aMPV detection because of their high analytical sensitivity and specificity. RT-PCR and RT-qPCR assays commonly target conserved genomic regions such as the nucleoprotein gene for broad detection, whereas glycoprotein-based assays support subtype differentiation [127,128,129]. Nevertheless, viral genetic variability may impair primer or probe binding and reduce assay performance. More recently, next-generation sequencing (NGS) and nanopore technologies have enabled direct genomic characterization from clinical samples, improving molecular epidemiology and identification of emerging variants [130,131]. Their wider implementation remains limited by cost and technical complexity.

Serological assays are primarily used for flock surveillance and retrospective diagnosis. ELISA remains the most widely applied platform because of its scalability and cost-effectiveness, although assay performance depends substantially on antigen selection, with recombinant nucleoprotein-based systems generally providing broader sensitivity. Virus neutralization assays offer greater specificity but remain labor-intensive and poorly standardized. Interpretation is particularly difficult in vaccinated flocks because distinguishing vaccine-derived from field-induced antibodies is challenging; paired serum sampling may improve interpretation [130].

Additional techniques including immunohistochemistry (IHC), immunogold labeling, and antigenic differentiation assays support localization of viral antigens within tissues and infected cells. These methods are especially useful in pathogenesis investigations and confirmation of respiratory epithelial tropism but are generally less sensitive than molecular approaches and depend on optimal tissue preservation.

Despite substantial advances in diagnostic technologies, important limitations remain, including short shedding duration, subtype diversity, co-infections, and interference from vaccination. In endemic production systems, widespread seropositivity may coexist with active viral circulation, complicating interpretation. Therefore, accurate diagnosis of aMPV requires integrated application of optimized sampling, molecular detection, serology, virus isolation, and genomic characterization to support both clinical diagnosis and epidemiological investigations [132,133,134,135,136,137].

## 6. Control Challenges of aMPV

Vaccination, together with strict biosecurity implementation, remains a cornerstone for the prevention and control of aMPV infection in commercial poultry systems. Effective control programs reduce respiratory disease severity, mortality, production losses, declines in egg production, and deterioration of egg quality associated with aMPV infection. In parallel, biosecurity measures—such as avoiding multi-age production systems, restricting inter-farm movement, implementing appropriate sanitation protocols, and minimizing environmental stressors affecting the respiratory tract—play a critical role in limiting viral transmission and reducing susceptibility to secondary bacterial infections.

Vaccination against aMPV has substantially reduced the clinical and economic impact of the disease in commercial poultry worldwide. However, unlike several other avian viral diseases, aMPV vaccination primarily aims to reduce clinical severity, viral shedding, secondary bacterial complications, and reproductive losses rather than induce sterilizing immunity. Consequently, vaccinated flocks may still support limited viral replication and environmental circulation under field conditions [138,139,140,141].

The first commercially relevant live attenuated turkey TRT vaccines were developed in Europe in the late 1980s following serial passage attenuation of subtype A viruses, particularly the UK/3B/85 strain [138]. These vaccines provided satisfactory protection against homologous subtype A challenge and were rapidly adopted in European turkey production systems [141]. During the early vaccination period in the United Kingdom, subtype A prevalence declined, while subtype B viruses subsequently emerged and became predominant in several poultry-producing regions [142,143]. Molecular epidemiological studies in Italy further demonstrated genetic divergence among circulating subtype B strains, particularly within glycoprotein genes, suggesting that prolonged immune pressure associated with widespread vaccination may contribute to antigenic drift, subtype replacement, and partial immune escape [143,144,145,146].

Currently used commercial vaccines are predominantly based on subtype A and subtype B viruses, while subtype C vaccines were historically developed mainly in North America, where subtype C circulation was largely associated with turkey production systems [146]. Experimental studies indicate substantial cross-protection between subtype A and subtype B vaccines, although protection levels may vary depending on strain differences, administration method, and host immune status [142]. Partial cross-protection against subtype C has also been observed following subtype A or B vaccination, although reciprocal protection appears less efficient [146,147].

Live attenuated vaccines are typically administered via coarse spray, drinking water, or oculo-nasal routes during early life in broilers and turkey poults to induce local respiratory immunity at the primary site of infection [148,149,150]. These vaccines stimulate mucosal immune responses characterized by activation of CD4+ and CD8+ T lymphocytes in the tracheal mucosa and Harderian glands, alongside local IgA production, which is critical for early protection [151,152]. In broilers, a single vaccination during the first week of life is often sufficient due to the short production cycle [153,154,155,156,157]. In contrast, long-lived birds such as breeders and commercial layers generally require a prime–boost strategy, typically involving live vaccination followed by an inactivated oil-emulsion booster prior to lay to enhance systemic immunity and protect reproductive performance [93,94,118,154].

In Egypt, several imported commercial vaccines are incorporated into vaccination programs for breeders, layers, broilers, and turkeys within intensive poultry systems. Commonly used products include NEMOVAC^®^, Poulvac TRT^®^, VAXXON SHS^®^, AVIFFA RTI^®^, HIPRAVIAR TRT^®^, and TUR-3^®^. Most of these vaccines are derived from subtype A or B strains, reflecting global vaccine development patterns and the absence of confirmed endemic subtype C circulation in Egypt.

Despite widespread vaccination, field investigations from Europe, the Middle East, and South America have confirmed continued circulation of subtype B viruses in vaccinated poultry populations [157,158,159,160,161]. Molecular studies in Italy and Israel identified amino acid substitutions in G and SH glycoproteins of circulating field strains compared to vaccine strains, which may contribute to reduced vaccine effectiveness and viral persistence [158]. Similar findings in Egypt have revealed genetically diverse subtype B viruses in vaccinated flocks, indicating ongoing viral evolution under field conditions [159].

Vaccination outcomes are further influenced by multiple epidemiological and operational factors, including incomplete flock coverage, improper vaccine administration, maternal antibody interference, high poultry density, and variable biosecurity levels [154,160]. Technical issues such as uneven spray distribution, incorrect drinking-water vaccination, concurrent respiratory infections, and inadequate vaccine handling may reduce vaccine take. Additionally, live vaccine strains may spread horizontally under field conditions [158], and repeated replication in susceptible hosts may contribute to genetic changes, raising concerns regarding reversion to virulence or vaccine-derived evolution [159,160,161,162,163].

Interactions with other respiratory vaccines further complicate field vaccination strategies. IBV vaccines may transiently interfere with aMPV vaccine replication due to competition for respiratory epithelial cells, potentially reducing local immune stimulation [161]. NDV vaccines generally show limited interference, although transient effects on replication have been reported [162,163]. Concurrent vaccination programs involving aMPV, NDV, and IBV therefore show variable immunological outcomes under commercial conditions [161,164]. Maternal antibodies may also reduce replication of live vaccines, particularly in turkeys with high antibody titers [165].

In response to limitations of conventional vaccines, several next-generation platforms have been explored experimentally. Recombinant vector vaccines based on fowlpox virus or Newcastle disease virus expressing aMPV glycoproteins F and G have shown promising protective efficacy and reduction in viral shedding [166,167,168]. Additional approaches, including DNA vaccines, recombinant subunit vaccines, virosomal formulations, and genetically modified attenuated mutants, have demonstrated immunogenic potential in experimental settings [169]. Deletion of accessory genes such as SH has also been explored as a strategy to develop genetically stable attenuated vaccine candidates with reduced reversion risk [170].

Overall, while vaccination remains essential for controlling aMPV-associated disease, it does not fully prevent viral circulation in densely populated poultry systems. Instead, vaccination acts both as a control tool and as a selective ecological pressure influencing viral evolution. In endemic settings such as Egypt, the coexistence of vaccinated commercial farms, partially vaccinated systems, backyard poultry, live bird markets, and wildlife interfaces facilitates ongoing viral circulation and genetic diversification. Therefore, long-term control requires integrated strategies combining optimized subtype-matched vaccination, strict biosecurity, improved vaccine application, and continuous molecular surveillance to monitor emerging variants and maintain vaccine–field strain compatibility.

## 7. Discussion: Beyond Conventional Views of aMPV Epidemiology in Africa and the Middle East

The evidence synthesized in this review suggests that the current understanding of avian aMPV may underestimate both its epidemiological importance and ecological complexity, particularly in Africa and the Middle East. Although aMPV has traditionally been framed as a respiratory pathogen of commercial poultry [7,35,49], the available literature increasingly indicates that its persistence cannot be fully explained through farm-level transmission alone. Instead, disease emergence appears to result from interactions among production structure, viral adaptation, host diversity, and environmental connectivity [24,25,54,95].

A recurring pattern identified across the reviewed studies is the disproportionate concentration of available evidence in regions with established surveillance capacity. Reports from North Africa and the Middle East remain highly uneven, with molecular confirmation concentrated in countries maintaining active surveillance programs, whereas large geographic areas remain underrepresented [8,77,96]. This raises an important interpretative challenge: the observed geographic distribution of aMPV likely reflects where testing is performed rather than where the virus truly circulates. Consequently, current maps of subtype occurrence should be interpreted as indicators of surveillance intensity rather than definitive representations of epidemiological boundaries.

Another important insight concerns the structure of poultry production itself. Poultryg control frameworks frequently treat commercial farms as isolated epidemiological units; however, this assumption appears increasingly difficult to support in regions where poultry production systems are highly interconnected. The coexistence of intensive farms, backyard holdings, live bird markets, and informal poultry movement may create continuous opportunities for viral exchange and respiratory pathogen maintenance [54,72,74,83,84,105,106]. Under these conditions, local elimination becomes difficult even when farm-level control measures are implemented successfully.

This broader perspective also changes how vaccination outcomes should be interpreted. Continued detection of field strains in vaccinated populations does not necessarily indicate vaccine failure. Rather, it may reflect a mismatch between the objectives of vaccination and the realities of endemic production environments. Current vaccination strategies primarily reduce disease expression and production losses but may exert limited influence on long-term transmission dynamics [23,141,165]. Therefore, epidemiological success should not be judged exclusively by reductions in clinical disease. Evidence from Egypt and Europe further suggests that vaccine-related evolutionary processes and field circulation of vaccine-like strains require cautious interpretation and do not alone explain persistence [89,143,146,158,159].

The predominance of subtype B across multiple settings in Africa and the Middle East further raises unresolved questions regarding viral adaptation. While immune pressure has frequently been proposed as a driver of subtype replacement [23,146], available evidence remains insufficient to support a direct causal relationship. Alternative mechanisms, including ecological fitness, production practices, host population structure, founder effects, and transmission efficiency—may collectively influence subtype persistence and geographic expansion [46,47,75,77,82,94]. This distinction is important because simplified evolutionary explanations may limit development of more effective control approaches.

The role of wildlife represents another area where interpretation should remain cautious. Detection of aMPV genetic material in wild birds has expanded considerably across multiple continents and host species [17,18,55,56,57,58,59]; however, ecological significance remains uncertain. Presence of viral RNA does not establish reservoir competence, sustained circulation, or meaningful contribution to transmission. A more useful framework may be to view wildlife as components of broader ecological networks rather than assuming direct epidemiological responsibility [55,59,64,94].

Egypt provides an illustrative regional example of these challenges. Its poultry sector combines high production density with ecological interfaces created by migratory pathways, wetlands, and extensive backyard production [70,71,72]. Molecular and serological investigations demonstrated progressive transition from historical serological evidence to endemic subtype circulation and, more recently, detection of subtype B across wildlife–poultry interfaces [86,87,88,89,90,91,92,93,94]. This overlap creates conditions where conventional epidemiological separation between domestic and environmental systems becomes increasingly difficult. Similar configurations may exist elsewhere in Africa and the Middle East but remain insufficiently characterized.

Collectively, this review supports a shift from viewing aMPV as an isolated production disease toward understanding it as an ecological and evolutionary phenomenon shaped by interactions across biological and production interfaces. Such a perspective may better explain persistent circulation patterns and provide a more realistic conceptual basis for future investigation of aMPV in Africa and the Middle East [8,24,25,54,55,95].

## 8. Conclusions

aMPV remains an important and increasingly recognized respiratory pathogen of poultry, with substantial implications for productivity, animal welfare, and disease control. Across Africa and the Middle East, available evidence supports widespread circulation of the virus, with subtype B predominating in most investigated settings and transmission occurring primarily through horizontal spread. The epidemiology of aMPV appears increasingly complex, involving interactions among commercial poultry, backyard flocks, live bird markets, wild birds, and environmental interfaces. Despite growing awareness, aMPV remains comparatively underrecognized and underdiagnosed in routine veterinary investigations because respiratory disease outbreaks are frequently attributed to more intensively monitored pathogens such as AIV, NDV, IBV and bacterial co-infections. Consequently, the true epidemiological burden of aMPV is likely underestimated. Future efforts should move beyond passive outbreak investigation and incorporate aMPV into routine laboratory diagnostic and surveillance programs. Expanded sampling strategies should include broilers, layers, breeders, backyard poultry, and wild birds, alongside environmental surveillance through water, litter, and interface-associated samples collected from wetlands, migratory sites, and poultry-dense production systems. Longitudinal studies with repeated sampling across production cycles should be prioritized and integrate molecular and serological approaches by combining RT-PCR, sequencing, phylogenetic analysis, antibody profiling, and vaccination history. Strengthened genomic surveillance and regional data sharing will be essential to improve understanding of viral evolution, vaccine–field strain relationships, and development of more effective regionally adapted control strategies.

## Figures and Tables

**Table 1:** Available avian metapneumovirus (aMPV) detections in Africa and the Middle East by country, subtype, host species, age, and diagnostic tool.

Country	References	Detection Year	Host Species	aMPV Subtype	Age (Range)	Type of Detection
**South Africa**	[1]	1978	Turkeys	A	NR	Virus isolation; indirect detection: serologic
**Algeria**	[76]	2012–2013	Turkeys (meat)	B	NR	Direct: molecular
**Egypt**	[86]	1997	Broilers	NR	NR	Indirect: serologic
	[87]	2008	Turkeys (meat)	NR	3–6 mo	Direct: molecular + indirect: serologic
	[88]	2013	Turkeys (meat)	A	3 wk	Direct: molecular
	[89]	2015	Turkeys	B	NR	Direct: molecular
	[90]	2017	Chickens (broilers)	B	NR	Direct: molecular
	[91]	2019	Chickens (broilers)	A and B	NR	Virus isolation; direct detection
	[69]	2024–2025	Chickens (broiler breeders)	B	16, 35 wk	Direct: molecular + indirect: serologic
	[93]	2018	Layers, broilers and ducks	NR	NR	Indirect: serologic
**Iran**	[102]	2016–2020	Chickens (broilers)	B	NR	Direct: molecular
	[103]	2009–2010	Chickens (broilers, breeders)	NR	>6 wk	Indirect: serologic
	[104]	2012–2013	Chickens (broilers)	NR	NR	Direct: molecular + indirect: serologic
	[99]	2018–2019	Chickens (broilers)	NR	NR	Direct: molecular + indirect: serologic
	[101]	2016–2018	Chickens (broilers)	B	NR	Direct: molecular
	[105]	2016	Chickens, turkeys (meat), wild birds	B	NR	Direct: molecular
	[100]	2014–2015	Turkeys (meat)	B	NR	Direct: molecular
**Iraq**	[96]	2018–2019	Chickens (broilers)	B	3–6 wk	Direct: molecular
	[97]	2021–2022	Chickens (broilers)	NR	6–8 wk	Indirect: serologic
**Israel**	[109]	2002–2004	Turkeys (meat)	A and B	NR	Direct: molecular
	[109]	2002–2004	Chickens (broilers, breeders)	NR	NR	Direct: molecular
	[110]	2006–2007	Turkeys (meat), chickens (broilers)	A and B	4 wk	Direct: molecular + indirect: serologic
**Saudi Arabia**	[111]	2007–2008	Chickens (broilers)	NR	11 wk	Indirect: serologic
	[112]	2019	Backyard chickens	NR	NR	Indirect: serologic
**Oman**	[106]	2012	Backyard chickens	B	NR	Direct: molecular
**Jordan**	[113]	2004–2005	Chickens (broilers, layers)	B	4 wk	Direct: molecular + indirect: serologic
	[114]	2005–2007	Chickens (broilers)	NR	NR	Direct: molecular
**Yemen**	[115]	NR	NR	NR	NR	Indirect: serologic
**Tunisia**	[74]	2015–2019	Chickens (broilers)	B	4 wk	Direct: molecular
	[75]	2018–2020	Chickens (broilers)	B	NR	Direct: molecular
**Turkey**	[107]	2017–2018	Chickens (broilers)	B	NR	Direct: molecular
	[108]	2017	Chickens (broilers, layers)	NR	NR	Molecular detection + virus isolation
**Morocco**	[77]	2020–2021	Chickens (broilers)	NR	NR	Indirect: serologic
	[78]	NR	Chickens (broilers)	A and B	23 d	Direct: molecular
	[79]	2022	Chickens (broilers)	B	NR	Direct: molecular
**Nigeria**	[80]	2008	Chickens, turkeys	A and B	NR	Direct: molecular
	[81]	NR	Chickens	B	NR	Direct: molecular
	[82]	NR	Chickens	B	NR	Direct: molecular
**Ethiopia**	[83]	NR	Chickens (breeders)	B	NR	Direct: molecular
	[84]	2021	Chickens	NR	NR	Direct: molecular

Direct detection includes molecular detection and/or virus isolation. Indirect detection refers to serological evidence (antibody detection). NR, Not Reported; wk, weeks; mo, months.

## Data Availability

No new data were created or analyzed in this study. Data sharing is not applicable to this article.
